# Supplementary material for: A Modified Wet Transfer Method for Eliminating Interfacial Impurities in Graphene
Source: Nanomaterials (Basel). 2023 Apr 27;13(9):1494. doi: 10.3390/nano13091494 (PMC10179892; doi:10.3390/nano13091494)
Supplement: Supplementary file 1 [file nanomaterials-13-01494-s001.zip › nanomaterials-2359810-supplementary.pdf]

Figure S1.

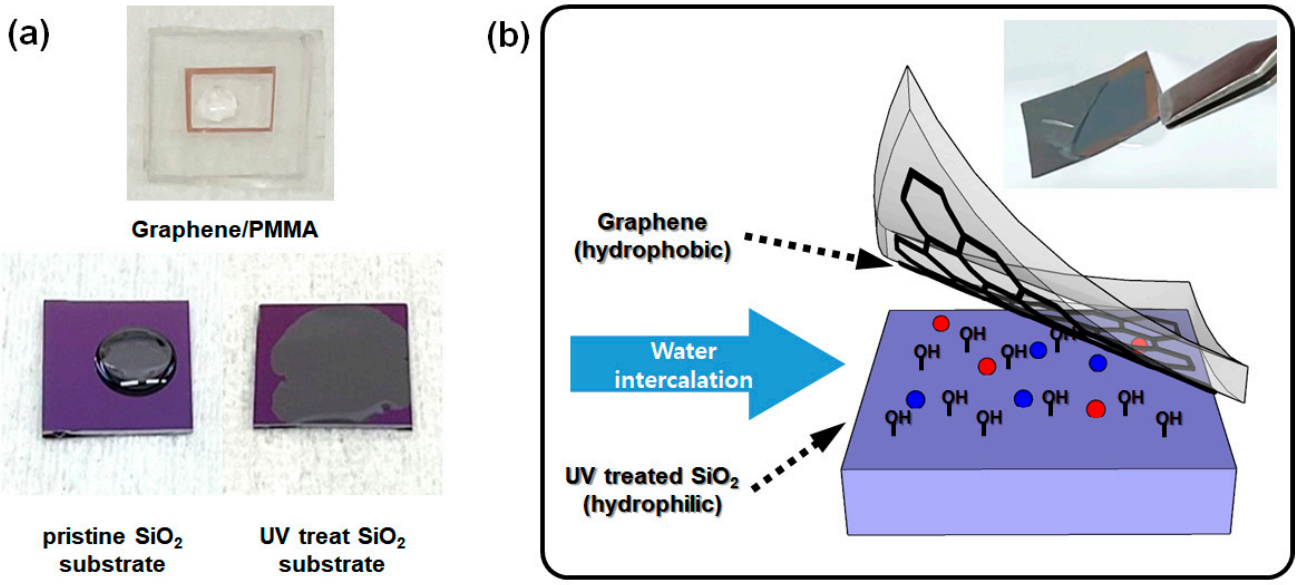

Figure S1. Hydrophobic graphene and UV-treated hydrophilic SiO_2 substrate. A schematic illustration of water intercalation between the interface of hydrophobic and hydrophilic surfaces. The inset shows the experimental result.

Figure S2.

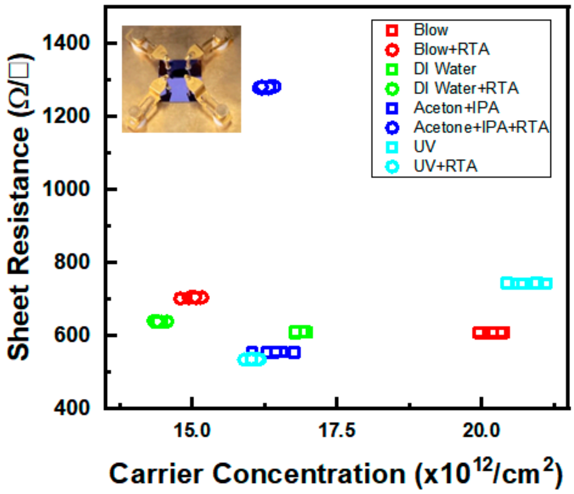

Figure S2. Carrier concentration vs. sheet resistance of graphene using various substrate treatments. The substrate treatments include nitrogen blow, DI water sonication for 40 min, acetone sonication for 20 min, isopropyl alcohol sonication for 20 min, and UV treatment. The square symbol indicates the original treatment, while the circle symbol represents the same treatment combined with RTA annealing. The inset displays the sample loading in 4-point Hall effect measurement.

Figure S3.

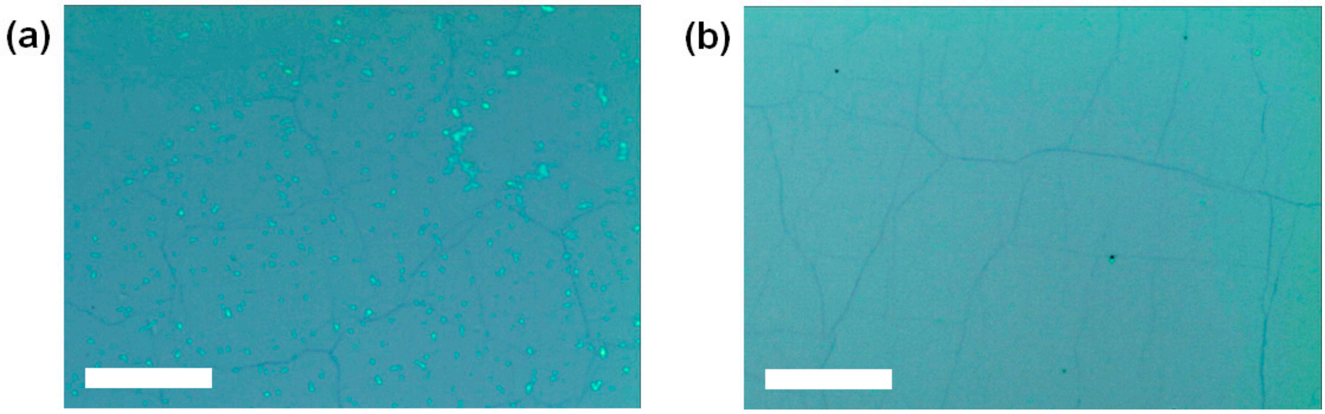

Figure S3. OM image of (a) conventional wet transferred graphene, and (b) retransferred graphene. The scale bar is 50 μm.

Figure S4.

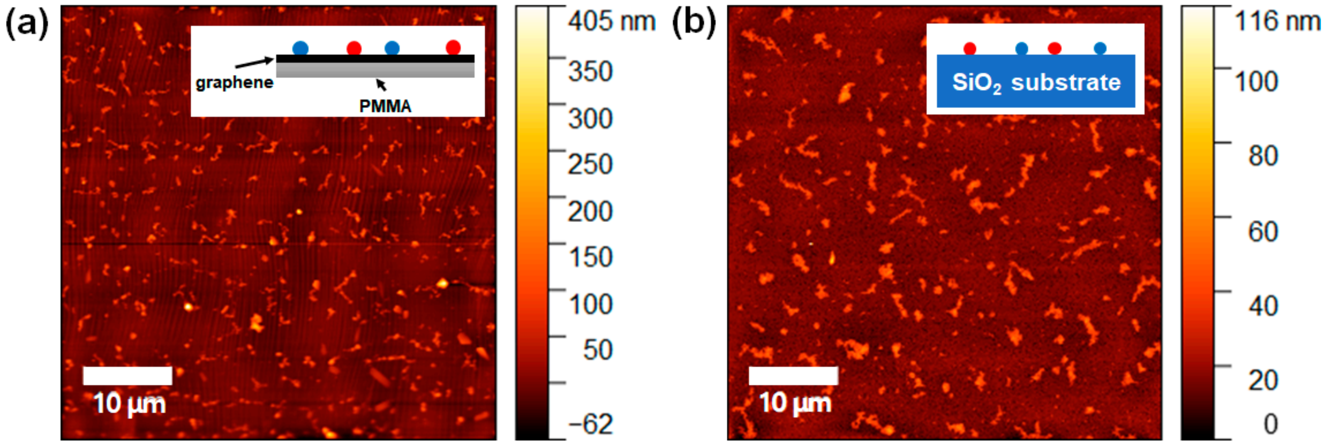

Figure S4. AFM topography of impurity distributions (a) on the graphene surface measured on wet etched PMMA/graphene during the transfer process, and (b) the temporary substrate after DI water-induced delamination during the retransfer process.

Figure S5.

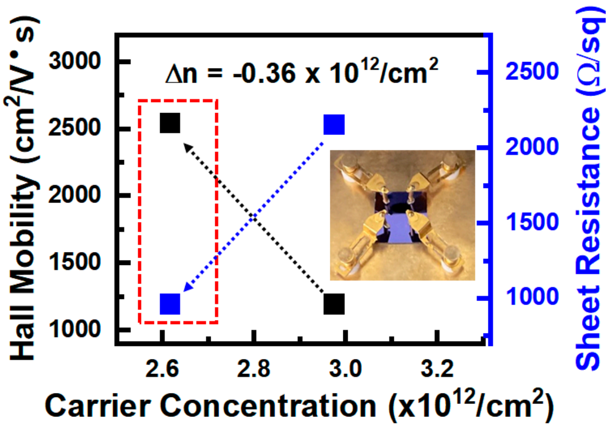

Figure S5. Sheet resistance and Hall effect mobility vs. carrier concentration changes of the transferred and the retransferred graphene samples. The change of each value is denoted with a dashed arrow from the transferred graphene to the retransferred graphene. The inset shows the carrier concentration difference (Δn) and the 4-point Hall effect measurement.
